# A Narrative Review of Q Fever in Europe

**DOI:** 10.7759/cureus.38031

**Published:** 2023-04-23

**Authors:** Magdalini Christodoulou, Foteini Malli, Konstantinos Tsaras, Charalambos Billinis, Dimitrios Papagiannis

**Affiliations:** 1 Department of Nursing, University of Thessaly, Larissa, GRC; 2 Department of Microbiology and Parasitology, Faculty of Veterinary Science, University of Thessaly, Karditsa, GRC

**Keywords:** narrative review, seroprevalence studies, q fever, europe, coxiella burnetii

## Abstract

*Coxiella burnetii*, the causative agent of Q fever, causes abortions in animals. Its effects on humans and the management of Q fever in certain conditions like pregnancy are undetermined. The World Health Organization has estimated that zoonotic diseases cause around one billion cases of infections and millions of deaths globally each year. It is worth noting that many emerging infectious diseases currently being reported worldwide are zoonoses. We reviewed studies reporting on Q fever prevalence and incidence in Europe. Articles from 1937 to 2023 with the following terms “*Coxiella burnetii and Europe and Q fever, and seroprevalence studies*” were identified in the PubMed database and reports by organizations such as the European Centre for Disease Prevention and Control (ECDC). We included randomized and observational studies, seroprevalence studies, case series, and case reports. According to the ECDC in 2019, 23 countries reported 1069 cases, the majority of which were classified as confirmed cases. The number of reports per 100,000 inhabitants in the EU/EEA was 0.2 for 2019, the same as the previous four years. The highest report rate (0.7 cases per 100,000 population) was observed in Spain, followed by Romania (0.6), Bulgaria (0.5), and Hungary. Considering the typically asymptomatic nature of Q fever infection, it is imperative to strengthen the existing systems to promote the rapid identification and reporting of Q fever outbreaks in animals, particularly in cases of abortion. It is also essential to consider the facilitation of early information exchange between veterinarians and public health counterparts to ensure the timely detection and prevention of potential zoonotic events, including Q fever.

## Introduction and background

*Coxiella burnetii* is an obligate intracellular Gram-negative bacterium and is the causative agent of Q fever, which is an occupational zoonosis with high infectivity and worldwide distribution except in New Zealand [1]. *C. burnetii* is a Gram-negative, strictly intracellular, pleomorphic bacterium ranging in size from 0.2 to 0.5 μm in width and 0.4-1.0 am in length. It belongs to the domain Bacteria, phylum Proteobacteria, class Gammaproteobacterial, order Legionellales, family Coxiellaceae, genus Coxiella and species *C. burnetii* [2]. It affects many species of domestic and wild animals which remain asymptomatic [3]. In humans, Q fever can cause severe clinical symptoms and even death. Even though it is considered an occupational zoonotic disease, cases have been recorded in humans without contact with animals. In many countries, it is not a notifiable disease, and as a result, the data are drawn either from confirmed outbreaks of infections or the National Reference Centers of the respective states. However, several cases are reported every year in Mediterranean countries. From 1999 to 2004, there were 18 reported outbreaks of Q fever from 12 different countries [4]. 

In 1937, Edward Holbrook Derrick suggested studying a feverish sickness that affected 20 out of 800 slaughterhouse laborers in Brisbane, Queensland, Australia [5]. This was the earliest recorded account of the disease, whose cause was unknown then. Due to the absence of an obvious etiology and a common infectious agent, he named the disease "Q fever" which stands for "Querry Fever". Although he was not able to isolate the pathogen responsible for the disease, he sent samples to Frank Macfarlane Burnet and Mavis Freeman. They were able to replicate the disease in guinea pigs, mice, and monkeys and observed large quantities of small bacilli in the vacuoles of the infected animals' spleen sections after Giemsa staining. These bacilli appeared similar in morphology to rickettsiae [6]. During that period, Herald Cox and Gordon Davis, who worked at the Rocky Mountain Laboratory in Montana, USA, separately identified a novel contagious agent in ticks gathered from Nine Mile Creek, Montana [7]. The agent exhibited properties similar to rickettsia.

A laboratory worker accidentally infected with this new organism developed symptoms almost identical to those of Q fever affecting Australian slaughterhouse workers, suggesting a common infectious agent [8,9]. Further studies in mice confirmed that these two newly discovered pathogens are the same pathogen [10]. Due to its Rickettsia-like properties, the pathogen was originally designated as *Rickettsia burneti.* In 1943, Cornelius B. Philip suggested establishing a new genus comprising only one species based on observable characteristics. He named the species "Coxiella burnetii" as a tribute to the contributions of Cox and Burnet in discovering the pathogen responsible for Q fever. [7]. Q fever has been recorded in most countries of the world. First, in 1955, Kaplan and Bertagna described the geographic distribution of Q fever [10]. The underreporting of Q fever cases is a common occurrence, particularly in countries that were not previously endemic for the disease. This poses a significant issue for public health, as it can have serious implications on both animal and human health. It is important to increase awareness and surveillance of the disease to accurately monitor and prevent its spread [11].

## Review

Materials and methods

We searched PubMed for studies reporting on Q fever prevalence and incidence in humans. The World Health Organization (WHO) Regional Office for Europe (WHO/Europe) is one of WHO’s six regional offices around the world. It serves the WHO European Region, which comprises 53 countries, covering a vast geographical region from the Atlantic to the Pacific Ocean selected a randomized representative sample of 12 countries from 53 total members WHO Regional Office for Europe through the Open Epi Random Program. Published articles for the present narrative review have been chosen by searching on accredited sites “PubMed” and official government sites such as public health organizations. The keywords we used for each country include “Coxiella burnetii; Europe, Q fever, seroprevalence studies". We used mainly articles in the English language that were published in international literature and especially in PubMed library. We aimed to provide data on the incidence and prevalence of Q fever in the Europe region.

Q fever in European countries

Greece

In Greece, *C. burnetii,* the etiological agent of Q fever, was isolated for the first time by Caminopetros during the Second World War, when epidemics of Q fever broke out in Greek and Italian soldiers [12]. In 1990, a study done by Alexiou-Daniil, in Northern Greece showed that 4.7% of 3686 patients with "atypical pneumonia" had antibodies against *C. burnetii *antigens [13]. In the period 1989-1993, 98 cases of Q fever were reported to the Laboratory of Parasitology, Zoonoses, and Geographical Medicine of Heraklion, Crete; 73.5% were men and they were recorded between January and June, and 35.4% had come into contact with unpasteurized dairy products. The main clinical symptoms were fever (91.7%) and respiratory symptoms (88.5%), while hepatitis was present in 52%. Eleven patients presented with neurological symptoms, and two patients (2.1%) had a skin rash [14]. In 2013, an extensive study of 5397 samples from patients with suspected *C. burnetii *infection was performed, of which 685 (12.7%) were initially evaluated positively for acute Q fever [15]. The mean age was 55.3 years. Of the 489 recovery samples, 134 (27.4%) showed fourfold seroconversion and were considered laboratory-confirmed cases of acute Q fever. Pneumonia was the main clinical symptom, and climatologic conditions or environmental parameters appeared to influence the distribution of Q fever which agrees with previous studies and shows a clear relationship of the disease with temperature and southerly winds [15]. In 2004-2005 in northern Greece, in a study carried out on 850 samples of patients with suspected *C. burnetii* infection, 58 (6.8%) were diagnosed with acute Q fever based on detection of IgM antibodies, and 1-2 weeks after the onset of symptoms, 76% were diagnosed between January and May [16]. The male/female ratio was 2:1, and the mean patient age was 45 ±5 years. One patient had a history of valvular disease, three patients had a history of immunodeficiency, 40 patients had contact with animals (mainly sheep) (69%), 43 patients were residents of rural areas (74.2%), and 15 patients were residents of urban areas (25.8%) [16]. In Greece, scientific studies on animals show the existence of *Rickettsia* and *C. burnetii* in the Greek area [17]. The wide spread of Q fever in Greece as well as a new genotype of *C. burnetii* was found in the aortic valve of a patient with Q fever endocarditis [18].

Netherlands

The Netherlands recorded the largest Q fever epidemic, both in duration and extent. During the years 2007-2010, 4026 cases of acute Q fever were recorded (mainly men, and smokers, aged 40-60 years) of which only 3.2% came from the agricultural sector and 0.5% from the meat processing industry. Respiratory symptoms were the most common clinical symptoms and were observed in 62% of cases [19,20].

About half of the infected individuals developed complications, such as heart failure, and 95 of them died [21,22]. Sixty percent of acute Q fever cases were asymptomatic, some had an influenza-like illness, and some developed severe disease with pneumonia, hepatitis, meningitis, meningoencephalitis, pericarditis, myocarditis, rash, pancreatitis, orchitis, optic neuritis, or osteoarthritis after an incubation period of usually 1-3 weeks [23]. Studies showed that the clinical manifestations were related to the mode of transmission: pneumonia, for example, after airborne transmission, and granulomatous hepatitis after ingestion of high doses of bacteria. After acute infection, some patients who are usually immunosuppressed, pregnant, or transplanted experienced chronic fatigue syndrome after Q fever. Chronic Q fever occurs more often as endocarditis or as seen in the Netherlands as vascular infection. Less often osteoarticular infections occur, as well as chronic hepatitis and pericarditis [22,24]. The mortality rate for chronic Q fever varies from 5 to 50%, depending on clinical manifestations and treatment options [25]. Also, it is estimated that the actual number of cases reached 50000 according to the Dutch Institute for Public Health and the Environment [26]. The disease turned into a nightmare for the Dutch government which proceeded to implement prevention measures and a legislative framework that included:

• The voluntary vaccination of herds with the COVEVAX (CEVA) vaccine (October 16, 2008),

• The mandatory vaccination in units >50 animals in the red areas and a 45 km radius around them (April 20, 2009),

• The ban on the import-export of animals from contaminated farms (October 1, 2009),

• Mandatory vaccination in sheep and goat units, zoos, visitable farms, etc. (January 1, 2010),

• Mandatory monitoring and examination of milk from all goat units every two months (December 14, 2009),

• The mandatory slaughter of all positive animals. (December 16, 2009),

• The application of diagnostic algorithms 

As reported by the Dutch health authorities, the primary contributing factor to the outbreak was the marked intensification of goat farming. Notably, the number of goats had increased fourfold since 1995, surpassing 350,000 animals in total. To modernize operations and take advantage of advanced technological equipment, large-scale animal farms were established. Despite these advancements, these farms were found to harbor a high concentration of animals, averaging around 1000 per farm, and were situated close to densely populated urban areas. The rate of Q fever cases per 100,000 people in European Union countries was 0.36 in 2010. Among these cases, 81.3% were reported in the Netherlands, France, and Germany combined. These three countries had the highest number of confirmed cases that year [25].

During the previous decade, there have been significant alterations in the epidemiological trends of diseases affecting animals utilized for commercial purposes. This has resulted in the emergence of novel outbreaks and substantial epidemics. These transformations appear to be connected to modifications in the organizational and structural aspects of animal production and management. Intensive animal husbandry, and especially the transport of goats between farms, favored the spread of the bacterium between animals and its transmission to humans [21,27].

Cyprus

The first report of the disease was in 1951 [28]. In 1974-1975, 78 British soldiers contracted Q fever. The incidence was 1.51/1000 in 1974 and by 1975 had fallen to 0.73/1000 [29]. In 2006, a surveillance program was conducted in two phases, and the incidence of the disease was found to be 1.2/100000 inhabitants per year. Despite the high positive prevalence in both humans and animals, a generally dwindling number of confirmed cases was observed [30].

France

In France, Q fever is endemic and commonly found, and the rural population living in the Alps has the highest reported prevalence of the condition. About 30% of individuals living in villages in this region have tested positive for specific antibodies associated with Q fever [31]. However, in other regions of France, such as Cote d'Or and Marseille, the prevalence of Q fever is lower with mean rates of 4.4% and 5% respectively [32]. The National Reference Center (NRC) established in 1985 examined for the period 1985-2009 a total of 179794 blood serum samples of which 3.723 (30%) had acute Q fever and 1.675 had chronic Q fever. Out of all the individuals who were diagnosed with acute Q fever, only 9.4% were identified as farmers, whereas 29.8% resided in a rural location. Contact with sheep was identified as one of the risk factors associated with the transmission of the disease. As a result of the reduction in the rural population in France over the past few decades, the incidence of the disease has also been reported among the urban population. This is mainly due to sporadic exposure to infected animals or consumption of contaminated raw milk [33].

In 1987, 40 cases of acute Q fever were diagnosed in a psychiatric institution in Banon, France [34]. In 1996 in Briançon, France, a town of 1500 inhabitants, 29 cases of acute Q fever were recorded [35]. Between 2000 and 2009, 907 patients with Q fever were recorded living in Provence Alpes Côte d'Azur in the region where the NRC is based. Between 2002 and 2003, 101 cases of patients with acute Q fever were recorded in Chamonix, France [36].

In 2009, a slaughterhouse in Cholet recorded 50 confirmed cases of acute Q fever [37]. In 1996 in central France, a seroepidemiological study was conducted and 208 sera from 168 breeders and 40 veterinarians and paramedical staff were examined. Antibodies were detected in 78% of breeders and 3/12 veterinarians and 0/28 paramedical personnel. The results of the study show the need for close monitoring of high-risk populations, to prevent the spread of the disease and its progression into a chronic form, and the most common clinical form is hepatitis [38,39].

The monthly distribution of acute Q fever for the period January 2000-December 2009 was determined to have the highest number of recorded cases between April and September [40].

Italy

Reports of the disease have existed since World War II from outbreaks in soldiers, and during 1949-1955 throughout Italy [41]. In Italy, the prevalence in some areas reaches 6.1%. In the summer and autumn of 1993, a large outbreak was recorded in Vicenza in North-Eastern Italy with 58 cases due to the movement of large numbers of sheep to higher pre-alpine pastures. Most cases were male (male/female ratio: 2.8:1). Exposure to migratory flocks of sheep was a risk factor. Of 100 herds surveyed by serological testing, 30 were found to be infected with *C. burnetii* [42].

In 2003, 133 cases of acute Q fever were recorded in the Prevention Department, Azienda Sanitaria Locale (ASL) of Como, presenting with high fever, dry cough, arthralgia and fatigue, and atypical pneumonia. Of these, 59 individuals were inmates from local prisons (which are located close to pastures grazed by sheep that were examined and the prevalence of the disease was 34.2%), 37 prison officers, 33 people living in the area traveled by the herd and 4 were staff of the Veterinary Service [43].

Switzerland

The incidence of the disease is high in Switzerland and reaches 11% in urban areas [44]. The Swiss Federal Public Health Service records 30-90 cases of Q fever per year [45]. In the fall of 1983, eight cases of Q fever were simultaneously recorded at the Marigny Hospital in patients living in the Val de Bagnes region (Valais, Switzerland). The outbreak was related to the movements of flocks (800-900 sheep) from the alpine pastures to the South, crossing several villages of the Val de Bagnes [46,47].

Out of the 3,036 individuals who were examined, serological diagnosis confirmed Q fever in 415 people, comprising 240 men and 175 women. Of these cases, a majority (224 out of 415, or 54%) were asymptomatic. However, of the 191 patients who were diagnosed with acute Q fever and evaluated by physicians, more than 75% exhibited persistent fever, shivering, and headaches [48].

Germany

The first reports of the disease were in World War II when German soldiers were infected during their stay in Greece and Italy [49]. By 1992, 31 outbreaks of Q fever affecting more than 5000 people were recorded in what was then West Germany [44]. In 1992, an outbreak was reported in Berlin [50], in 1993 in Dortmund [51], and in 1994 in Dusseldorf [52]. The herds of sheep and goats near the river Rhine are the source of infection. Atypical pneumonia is the most common manifestation of the disease [4], while inhalation of contaminated air particles is the most common route of transmission [1].

United Kingdom

The disease is considered endemic throughout England and Wales. The initial documentation of Q fever infection in men dates back to 1949 in the United Kingdom [53]. Between 1967 and 1974, 59 cases were recorded per year, while 1995 saw an increase with 169 confirmed cases [54]. Between 1980 and 1996, eight cases were reported in the literature [7]. In the Plymouth study between 1972 and 1988 for two areas, one in southwest Devon (population 42000) and the other in east Cornwall (population 91000) 103 confirmed cases were reported [55]. In 1982, the PHLS Communicable Disease Surveillance Center recorded 29 cases in Wales in the general population [56]. Fourteen cases were recorded in southwest England among laboratory personnel after exposure to experimentally infected sheep [57]. In 1983, 25 cases were reported among Oxford postal workers [58]. Q fever endocarditis accounted for approximately 3% of all cases of endocarditis reposted in the UK between 1975 and 1981 [59]. In the year 1987, there were five recorded cases of Q fever among school students in the southwestern region of England. It was suspected that the source of the infection was the school's animals, which included poultry and goats [60,61].

In 1992, four cases were reported in the Isle of Wight among waste disposal workers [62]. Finally, in June 2006, 138 confirmed cases were recorded in slaughterhouse workers near Stirling in Scotland where, due to the large immigration influx since 2004, 48 involved migrant slaughterhouse workers and in particular 41 workers from Slovakia, 3 from Poland, 2 from Lithuania, and 2 from the Czech Republic [63]. Occupational employment was also studied as a risk factor, and a comparative study was done on 404 farmers and 395 police officers where it was found that the prevalence was three times higher in those with agricultural occupations [64].

During the period spanning from 2000 to 2015, a total of 904 instances of acute fever were documented in England and Wales. This figure indicates an average yearly occurrence of 0.09 cases per 100,000 individuals per annum (excluding outbreaks). It is noteworthy that this value is marginally less than the previously reported estimations of 0.1 to 0.35 cases per 100,000 individuals per annum for the period of 1975 to 1995 [65].

North Ireland

Q fever is endemic in Northern Ireland. From 1962 to 1989, 443 patients were diagnosed. In 1997, there was an increase in the incidence of the disease with 107 cases of which 47 came from the Antrim area, an area with many sheep with an average patient age of 40-49 years, and most of them male. The main clinical symptom is a respiratory infection. Between 1971 and 1974, a study examined 1,587 serum samples from patients with febrile illnesses. It was found that 86 individuals had antibodies against phase II of *C. burnetii*, indicating a prevalence of Q fever infection in the population. In another seroepidemiological study, 28% of patients were found to have antibodies against *C. burnetii*, suggesting a relatively high incidence of Q fever in the studied population [66]. In 1986, two laboratory-confirmed cases were reported in Northern Ireland [60]. In 1989, a total of 147 cases of Q fever were reported in the Midlands region [65]. An additional 47 cases were reported in the community of Northern Ireland [61].

Spain

Spain has a higher incidence of Q fever cases in comparison to other European countries, with specific rural regions reporting rates as high as 15.4%, while urban areas have rates of 8.8% [40]. The majority of diagnosed cases were reported in Northern Spain, particularly in the Basque and Navarre provinces, which have a greater concentration of livestock activities [67]. From 1981 to 1985, the Centro Nacional de Microbiología, Virología e Inmunología Sanitarias confirmed 249 cases of Q fever from different regions of Spain, including 234 cases of acute Q fever and 15 cases of chronic Q fever, with 14 cases diagnosed with endocarditis. Most acute cases were reported in men (77.1%) between the ages of 15 and 44, with atypical pneumonia being the most common clinical manifestation (75%), followed by febrile illness (18%). Liver involvement was documented in 7.4% and 19% of patients with pneumonia or febrile illness, respectively [68]. The Madrid region reported a significant number of cases, although this may have been influenced by the proximity of the referral center [3]. The disease was less common in the central and southern areas of the country. However, it was observed that the way the disease presented clinically differed in various regions of Spain. In the northern Basque region, pneumonia was the most frequent form of manifestation, while hepatitis was more predominant in Andalusia in southern Spain [69].

Between 1984 and 1985, Montejo Baranda and colleagues reported 130 cases of Q fever pneumonia [70]. The ratio of affected males to females was 3:1. The majority of cases (86.9%) occurred in individuals between the ages of 11 and 40 years who had regular or occasional contact with cattle, sheep, or goats. Consuming unpasteurized milk was also considered a potential risk factor for Q fever pneumonia [70]. A seasonal increase in cases between March and July was also observed [71]. The main clinical symptoms of the disease include fever, headaches, myalgias, and respiratory symptoms. Radiological findings were detected in 85 (65.4%) of the patients, while elevated levels of liver transaminases were recorded in 80 patients (61.5%) [1].

Russia

Between 1957 and 2019, 13,836 cases of Q fever were officially registered in the Russian Federation, with the highest incidence in 1957 and the lowest in 2008, and the majority of cases occurring in the Southern Federal District, particularly in the Astrakhan Region, followed by the North Caucasus and Central Federal Districts, with lower incidence rates reported in the Volga, North-Western, Siberian, and Ural Federal Districts, and no registration of Q fever in the Far Eastern Federal District [72]. According to literature reports, goats are the main reservoir of Q fever in Russia [73].

Bulgaria

The first cases of human Q fever in Bulgaria were reported in 1949 [11]. Extensive epidemiological and epizootological studies were conducted due to the consolidation of livestock farms in state facilities and large agricultural cooperative units, leading to a high concentration of animals, mainly cows and sheep. This situation created a large natural reservoir for the incubation, circulation, and preservation of *C. burnetii* in the wild. The incidence of *C. burnetii* infection in various parts of the country ranged from 6% to 100% in sheep, 5% to 31% in cattle, and 7% to 34% in goats. Tick infestation with *C. burnetii* was also found to be prevalent, reaching 26% in southwestern and 22% in northeastern Bulgaria [74].

The situation changed dramatically in the 1990s as large-state facilities and cooperative farms collapsed and the number of cows and sheep decreased, for example, sheep decreased from 8,000,000 in 1990 to 3,000,000 in 1997. As a result, ranchers began to raise goats for easily accessible food, and the number of goats increased from 430,000 in 1990 to more than 1,000,000 in 1997. In 1996-1997, 90% of 140 samples from goats and 73% of 118 sheep tested positive for *C. burnetii*. These changes also affected the occurrence and seasonality of human Q fever outbreaks in Bulgaria. These outbreaks of Q fever among farmers explain the sharp peak of Q fever incidence in 1985. An increase in cases was observed in the spring and summer months (March to August) with a peak in May and June in the 1980s. Risk factors were reported to include changes in fertilization techniques and contact with infected animals, mainly goats. Goats were found to be the most important source of *C. burnetii* infection for humans as they grazed daily (from March to October) and crossed roads in villages and small towns twice a day. The largest outbreak of Q fever was recorded in Panagyurishte, the central part of southern Bulgaria, in the 1990s, following an influenza epidemic (December 1992-January 1993). Between January and June of 1993, a second wave of an epidemic occurred, resulting in over 2,000 cases of acute influenza respiratory illness and bronchopneumonia. Of these cases, 589 were diagnosed with atypical pneumonia through radiography. Out of 500 individuals who had recuperated from Q fever, 60% were confirmed to have had the disease through serological testing. This indicates that Q fever played a major role in the epidemic [75].

While most Q fever patients were adults between the ages of 20 and 59, high positivity rates were also observed in children under six years old (19%) and patients aged 7-19 years (23%). Most of the patients did not engage in animal husbandry or process animal products. Serological testing of domestic animals revealed equivalent results for goats (26% of 969) and sheep (28% of 421). The highest number of human Q fever cases occurred from April to June, which corresponds to the period of goat calving or abortion. In April-June 1995, a new outbreak of Q fever was reported in Panagyurishte. As part of this outbreak, 89 patients with bronchopneumonia were admitted to the local hospital. Of these patients, 78% were found to be positive for Q fever, indicating a high prevalence of the disease in the affected population [2]. The study's findings indicate that Q fever is an endemic disease in the Panagyurishte region of Bulgaria, and it appears seasonally in the spring. In this region, goats are thought to be the main source of human infection. Seasonal outbreaks of Q fever have also been noted in other regions of Bulgaria, such as Ikhtiman, Elin Pelin, Stara Zagora, Blagoevgrad, Vratza, and Varna [75]. By 1997, five patients with chronic Q fever presenting as endocarditis had been confirmed through serological testing. Additionally, by 1997, five cases of chronic Q fever manifesting as endocarditis were serologically confirmed. Two more cases of Q fever endocarditis were serologically diagnosed from 1996 to 1997. In a study, it was found that 16 out of 18 pregnant women who miscarried had antibodies against phase II *C. burnetii* from MA. The titers of these antibodies ranged from 10 to 320, which suggests that the possibility of acute Q fever infection during pregnancy cannot be ruled out [74]. An investigation was conducted into a Q fever outbreak that occurred in the Western Bulgarian town of Botevgrad between May 1 and June 9, 2004, resulting in the identification of 220 cases, with 168 of them being from Botevgrad and the rest from neighboring towns. This outbreak was the most extensive one in the last two decades in Bulgaria, and it was unusual for Q fever to spread in an urban area. The outbreak was traced back to flocks of sheep and goats, which were deemed to be the most likely source of the infection [76].

Q fever is a common zoonotic disease in Bulgaria, which means that it can be transmitted from animals to humans. The primary mode of transmission is through inhaling infected aerosols. In Bulgaria, there has been a rise in both sporadic cases and outbreaks of Q fever over the past decade [77].

Table 1 shows the number of recorded cases of Q fever, per EU country, for the period of 2008-2020, as extracted from the Disease Surveillance Atlas of ECDC.

**Table 1:** Number of reported cases of Q fever, per country EE, for the years 2008-2020 Information from ECDC [78]

Country EU	2008	2009	2010	2011	2012	2013	2014	2015	2016	2017	2018	2019	2020	Total	%
Belgium	27	33	30	6	7	5	3	8	16	7	6	10	4	162	1.24
Bulgaria	17	22	14	12	29	23	15	15	17	28	45	36	103	376	2.79
Croatia	0	0	0	0	43	0	21	14	8	23	11	8	2	130	0.96
Cyprus	31	2	4	5	4	3	1	4	2	3	0	1	1	61	0,45
Czech Republic	0	0	0	1	1	0	0	1	2	0	1	1	1	8	0.06
Finland	2	1	5	0	0	5	0	3	2	4	2	2	0	26	0.19
France	0	0	286	228	5	158	209	250	251	194	172	156	96	2005	14.89
Germany	370	191	326	285	198	114	238	310	270	107	91	148	55	2703	20.08
Greece	3	3	1	3	11	11	15	10	9	4	13	14	4	101	0.75
Hungary	11	19	68	36	36	135	59	35	39	24	28	47	34	571	4.24
Ireland	10	17	9	4	5	0	0	4	6	2	0	2	2	61	0.45
Italy	0	0	0	0	0	0	0	0	3	7	1	6	0	17	0.12
Latvia	1	0	2	1	1	1	3	1	0	0	0	0	1	11	0.08
Luxembourg	0	0	0	0	0	0	0	1	0	0	0	0	2	3	0.02
Malta	0	0	0	0	0	2	0	0	0	0	2	1	0	5	0.037
Netherlands	1039	2354	504	80	63	20	26	20	14	22	18	16	7	4183	31.08
Norway	0	0	0	0	0	4	1	1	2	4	5	8	5	30	0.22
Poland	12	3	0	0	0	0	1	0	0	0	0	4	0	20	0.14
Portugal	12	14	13	5	26	21	25	20	17	48	36	32	22	291	2.16
Romania	3	2	7	6	16	24	21	3	32	46	22	109	12	303	2.25
Slovakia	0	0	0	0	0	0	1	0	0	0	2	1	5	9	0.06
Slovenia	0	0	1	0	1	1	3	1	1	3	1	6	1	19	0.14
Spain	119	34	69	33	58	75	77	97	249	333	313	332	170	1959	14.55
Sweden	7	5	11	5	2	3	2	4	3	3	7	10	1	63	0.46
United Kingdom	56	19	30	43	12	46	60	21	34	21	19	9	0	370	2.74
EU	1712	2719	1380	757	518	647	780	822	975	884	790	951	523	13458	

## Conclusions

Globalization has significantly increased the movement of people, animals, and goods across national borders, resulting in the global spread of zoonotic infections. These infections often can spread across borders from their place of origin, causing a significant economic impact on industries such as trade, commerce, tourism, and consumer confidence. Transboundary diseases can have devastating consequences on the economy. Q fever remains a neglected zoonosis in many developing countries of the world including countries of Europe region. Mandatory notification of Q fever in humans is an important surveillance strategy and has been recommended and should be continuing to occur in Public Health sectors. The cooperation and flow of information between veterinary and medical professionals, and vice versa, is important. The disease has been reported in all European countries but with a lower incidence or perhaps less diagnosed in northern countries. There are reports from Sweden, Finland, Poland, the Czech Republic, Slovakia, and Romania.

As Q fever infection in animals is typically asymptomatic, it is essential to improve systems for detecting and reporting outbreaks of Q fever, particularly in instances of abortion episodes. Communication channels between veterinarians and public health professionals should be enhanced to facilitate the early exchange of information about potential zoonotic events, including Q fever. Surveillance efforts in domestic ruminants should prioritize small ruminants to gain a more accurate understanding of the human exposure risk. Furthermore, raising awareness among farmers and veterinarians regarding *C. burnetii* infection in farmed ruminants and the risk factors for spillover to humans is crucial. To minimize shedding from infected animals, strategies such as culling pregnant animals, temporary breeding bans for Q fever, stamping out, identification and elimination of shedders, control of animal movements, and standstill measures should be considered. The multidisciplinary cooperation responsible for sharing and assessing signals of emerging zoonotic pathogens and informing the necessary parties within the Zoonoses surveillance must be integrated for the One Health approach in public health sectors.
